# Trehalose ameliorates oxidative stress-mediated mitochondrial dysfunction and ER stress via selective autophagy stimulation and autophagic flux restoration in osteoarthritis development

**DOI:** 10.1038/cddis.2017.453

**Published:** 2017-10-05

**Authors:** Qian Tang, Gang Zheng, Zhenhua Feng, Yu Chen, Yiting Lou, Chenggui Wang, Xiaolei Zhang, Yu Zhang, Huazi Xu, Ping Shang, Haixiao Liu

**Affiliations:** 1 Department of Orthopaedic Surgery, The Second Affiliated Hospital and Yuying Children’s Hospital of Wenzhou Medical University, 109, Xueyuanxi Road, Wenzhou 325027, China; 2 Department of Rehabilitation, The Second Affiliated Hospital and Yuying Children’s Hospital of Wenzhou Medical University, 109, Xueyuanxi Road, Wenzhou 325027, China

## Abstract

Oxidative stress-related apoptosis and autophagy play crucial roles in the development of osteoarthritis (OA), a progressive cartilage degenerative disease with multifactorial etiologies. Here, we determined autophagic flux changes and apoptosis in human OA and tert-Butyl hydroperoxide (TBHP)-treated chondrocytes. In addition, we explored the potential protective effects of trehalose, a novel Mammalian Target of Rapamycin (mTOR)-independent autophagic inducer, in TBHP-treated mouse chondrocytes and a destabilized medial meniscus (DMM) mouse OA model. We found aberrant p62 accumulation and increased apoptosis in human OA cartilage and chondrocytes. Consistently, p62 and cleaved caspase-3 levels increased in mouse chondrocytes under oxidative stress. Furthermore, trehalose restored oxidative stress-induced autophagic flux disruption and targeted autophagy selectively by activating BCL2 interacting protein 3 (BNIP3) and Phosphoglycerate mutase family member 5 (PGAM5). Trehalose could ameliorate oxidative stress-mediated mitochondrial membrane potential collapse, ATP level decrease, dynamin-related protein 1 (drp-1) translocation into the mitochondria, and the upregulation of proteins involved in mitochondria and endoplasmic reticulum (ER) stress-related apoptosis pathway. In addition, trehalose suppressed the cleavage of caspase 3 and poly(ADP-ribose) polymerase (PARP) and prevented DNA damage under oxidative stress. However, the anti-apoptotic effects of trehalose in TBHP-treated chondrocytes were partially abolished by autophagic flux inhibitor chloroquine and BNIP3- siRNA. The protective effect of trehalose was also found in mouse OA model. Taken together, these results indicate that trehalose has anti-apoptotic effects through the suppression of oxidative stress-induced mitochondrial injury and ER stress which is dependent on the promotion of autophagic flux and the induction of selective autophagy. Thus, trehalose is a promising therapeutic agent for OA.

Osteoarthritis (OA) is the most prevalent form of degenerative joint disease characterized by articular cartilage degeneration, joint pain and functional impairment, and affects millions of people worldwide.^1, 2^ The etiology of OA is multifactorial, but several pathological factors such as inflammatory cytokines, mechanical stress and senescence lead to increasing levels of reactive oxygen species (ROS).^3^ ROS plays a crucial role in OA by provoking oxidative stress, which induces mitochondrial injury and activates ER stress to trigger a cascade of apoptosis.^4, 5, 6, 7, 8, 9^ Tert-Butyl hydroperoxide (TBHP) is an exogenous inducer of oxidative stress with several advantages over H_2_O_2_ such as high stability and slow release.^10^ TBHP has been widely used for *in vitro* studies on OA.^10, 11, 12^

Autophagy is an intracellular catabolic mechanism through which cells remove dysfunctional organelles and macromolecules to protect against cellular stresses.^13^ Autophagy is initiated by the sequestration of cytoplasmic components in double-membrane vesicles termed autophagosomes, which later fuse with lysosomes and the contents are degraded by lysosomal proteases.^14^ This process is termed autophagic flux. Autophagy is closely associated with apoptosis in the pathogenesis of many degenerative diseases, and decreased autophagy has been reported in OA.^15, 16, 17, 18^ Intra-articular injection of rapamycin, a classical activator of autophagy, can delay cartilage degeneration.^19^ Cetrullo *et al.*^20^ found that autophagic flux was inhibited under oxidative stress in human chondrocytes. Therefore, we propose that the induction of autophagy and the restoration of autophagic flux might be a promising therapeutic approach for OA. Moreover, autophagy can selectively occur in certain conditions for the organelles such as the mitochondria,^21, 22^ ER,^23, 24^ and peroxisomes,^25^ among which mitophagy and reticulophagy are involved in the maintenance of mitochondria and ER homeostasis and contribute to the inhibition of apoptosis. Thus, selectively targeting autophagy might be effective for protecting chondrocytes from excessive apoptosis.

Trehalose, a natural disaccharide that presents in a diverse range of organisms including plants, bacteria, yeast and fungi, is a novel autophagy activator in many cells through mTOR independent pathway.^26, 27, 28^ Trehalose exerts cell protective effects under various stress conditions such as oxidative damage, dehydration and temperature changes.^29^ Potential mechanisms of trehalose induced anti-apoptosis effects include rescuing mitochondria dysfunction, suppressing ER stress and restoring autophagic flux.^26, 27, 30^ In addition, trehalose could activate BNIP3 to reverse arterial stiffening with age and BNIP3 is associated with the initiation of mitophagy and reticulophagy.^25, 31^ The protective effects of trehalose have been reported in several degenerative diseases, but not yet in OA.^27, 30, 32^ Therefore, in this study we investigated the effects of trehalose on apoptosis and autophagy in chondrocytes under oxidative stress and explored the potential mechanism. Furthermore, we evaluated the therapeutic potential of trehalose in a destabilized medial meniscus (DMM) mouse model of OA.

## Results

### Blocked autophagic flux and increased apoptosis in human OA and TBHP-treated mouse chondrocytes

**Figure 1:**
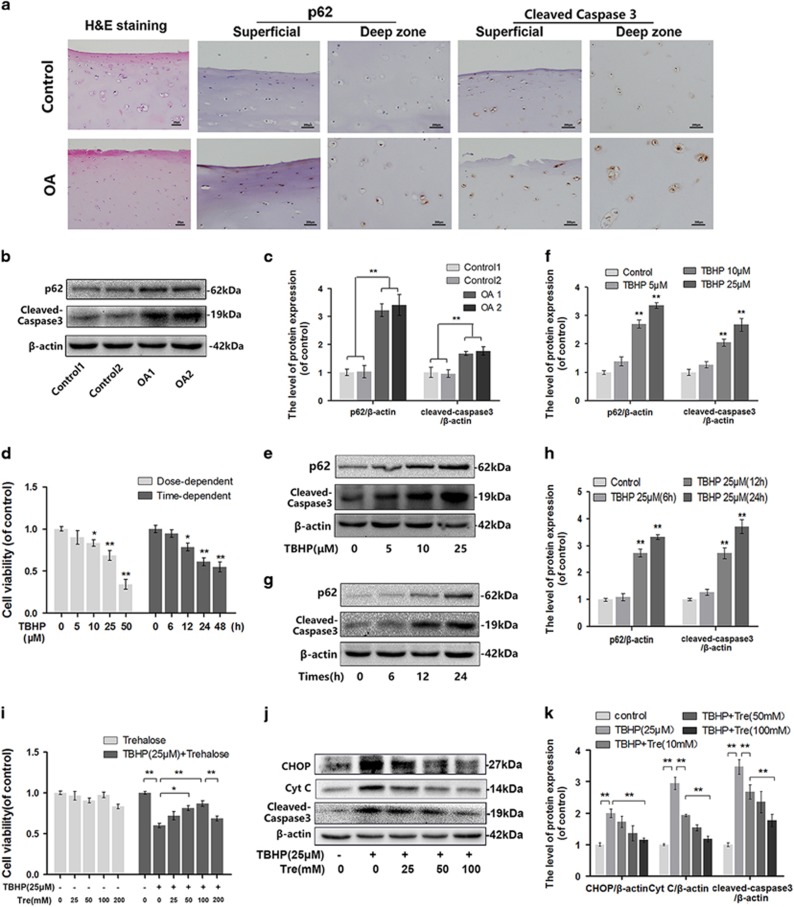
Trehalose inhibits TBHP induced apoptosis in chondrocytes. (**a**) Representative H&E staining and immunohistochemical staining of p62 and cleaved caspase 3 in superficial and deep zones of cartilage from controls and severe osteoarthritis patients. (bar: 200 *μ*m). (**b** and **c**) p62 and cleaved caspase 3 levels in chondrocytes derived from osteoarthritis patients and controls. (**d**) The viability of mouse chondrocytes after TBHP treatment. (**e** and **f**) p62 and cleaved caspase 3 levels in mouse chondrocytes treated with different concentrations of TBHP for 24 h. (**g** and **h**) p62 and cleaved caspase 3 levels in mouse chondrocytes treated with 25 *μ*M TBHP in different time points; (**i**) The viability of mouse chondrocytes after trehalose treatment alone or both trehalose and TBHP treatment. (**j** and **k**) The levels of CHOP, Cyt C and cleaved caspase 3 in mouse chondrocytes after TBHP treatment alone or both trehalose and TBHP treatment. All data represent mean±S.D. (*n*=5). **P<0.01, *P<0.05, *versus* Control

To investigate whether autophagic flux is aberrantly blocked in OA, we employed immunohistochemical analysis to compare p62 level in human cartilage obtained from OA subjects and controls. We found high accumulation of p62 in the cartilage from OA cartilage, accompanied by increased levels of cleaved caspase 3, a typical apoptosis marker (Figure 1a). Western blot analysis revealed higher levels of p62 and cleaved caspase 3 in the chondrocytes derived from OA subjects compared to control subjects (Figures 1b and c). The mouse chondrocyte phenotype was determined by toluidine blue staining and immunofluorescence of type II collagen (Supplementary Figure S1). By CCK8 assay and western blot analysis we found that TBHP decreased the viability of mouse chondrocytes (Figure 1d), and induced the accumulation of p62 and the cleavage of caspase 3 in a dose- and time-dependent manner (Figures 1e–h).

### Trehalose inhibits oxidative stress-induced apoptosis in chondrocytes by inhibiting mitochondria dysfunction and attenuating ER stress

The cytotoxic effects of trehalose on chondrocytes were determined by CCK8 assay. Trehalose treatment showed no significant cytotoxicity at concentrations up to 200 mM at 24 h, but trehalose pretreatment reversed TBHP-induced cytotoxicity at concentrations ≤100 mM (Figure 1i). TBHP-treated chondrocytes showed morphological changes including cell shrinkage, vacuole formation in the cytoplasm, and floating cells in the medium. However, these changes were attenuated with trehalose intervention (Supplementary Figure S2). In addition, Western blot analysis revealed that TBHP-induced increased levels of cleaved caspase 3, cytochrome C (Cyt C, marker of Mitochondria dysfunction) and C/EBP Homologous Protein (CHOP, marker of ER-stress) in chondrocytes were antagonized by trehalose pretreatment in a dose-dependent manner (Figures 1j and k).

### Trehalose induces autophagy and enhances autophagic flux in chondrocytes

**Figure 2:**
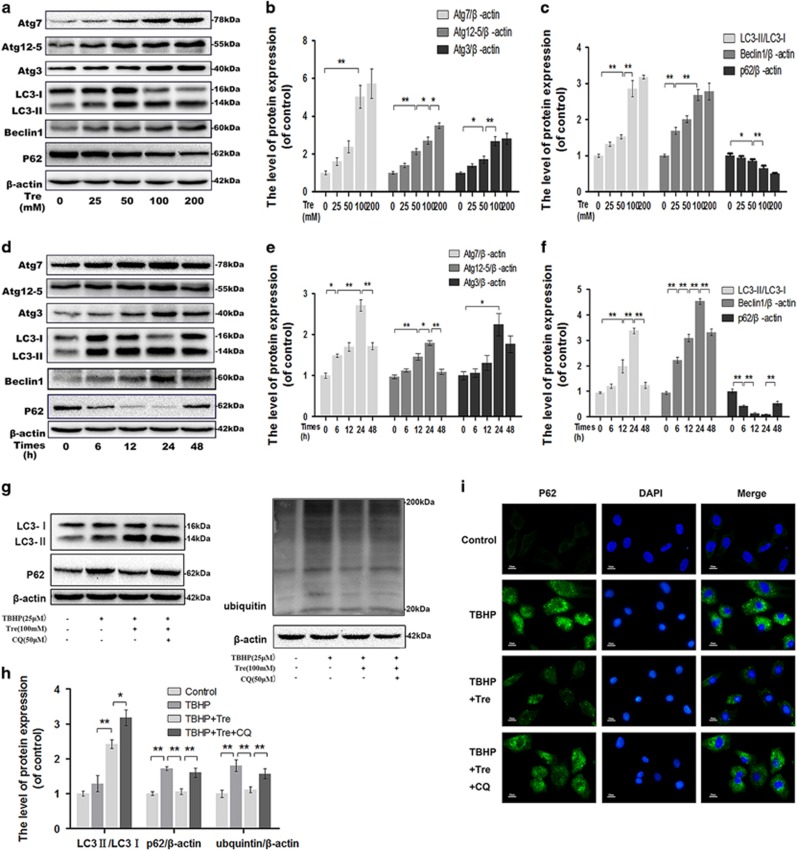
Trehalose induces autophagy in chondrocytes. (**a–f**) The chondrocytes were treated with 0, 25, 50, 100 or 200 mM trehalose for 24 h or 100 mM trehalose for 0, 6, 12, 24 or 48 h, and protein levels of Atg7, Atg12-5, Atg3, LC3, Beclin-1 and p62 were detected. **(g** and **h**) The chondrocytes were treated with TBHP, trehalose or CQ, and the levels of LC3, p62 and ubiquitin were detected. (**i**) p62 accumulation was examined by immunofluorscence staining (bar: 10 *μ*m). All data represent mean±S.D (*n*=5). **P<0.01, *P<0.05

Western blot analysis showed that several indicators of autophagy formation, including Atg3, Atg7, Atg12–5 and Beclin1, and LC3II/LC3I ratio increased in trehalose-treated chondrocytes in a dose-dependent manner. However, p62 level decreased after trehalose treatment, indicating that trehalose could enhance autophagic flux (Figures 2a–c). Next, we investigated the time-course of trehalose-induced activation of autophagy. Western blot analysis revealed that trehalose activated autophagy gradually, which peaked at 24 h (*P*<0.01), consistent with p62 degradation (Figures 2d–f).

### Trehalose reverses TBHP-induced blockage of autophagic flux

To confirm that trehalose induced autophagic changes in TBHP-treated chondrocytes, we blocked autophagic flux by using chloroquine (CQ), a classical autophagic flux inhibitor.^14^ TBHP treatment led to significantly increased levels of p62 and ubiquitin, but did not decrease the ratio of LC3-II/LC3-I. As expected, trehalose treatment reduced TBHP-induced accumulation of p62 and ubiquitin while increased the ratio of LC3-II/LC3-I, but these effects were inhibited by CQ (Figures 2g–i).

### Trehalose induces mitophagy and reticulophagy in chondrocytes and upregulates BNIP3 and PGAM-5

**Figure 3:**
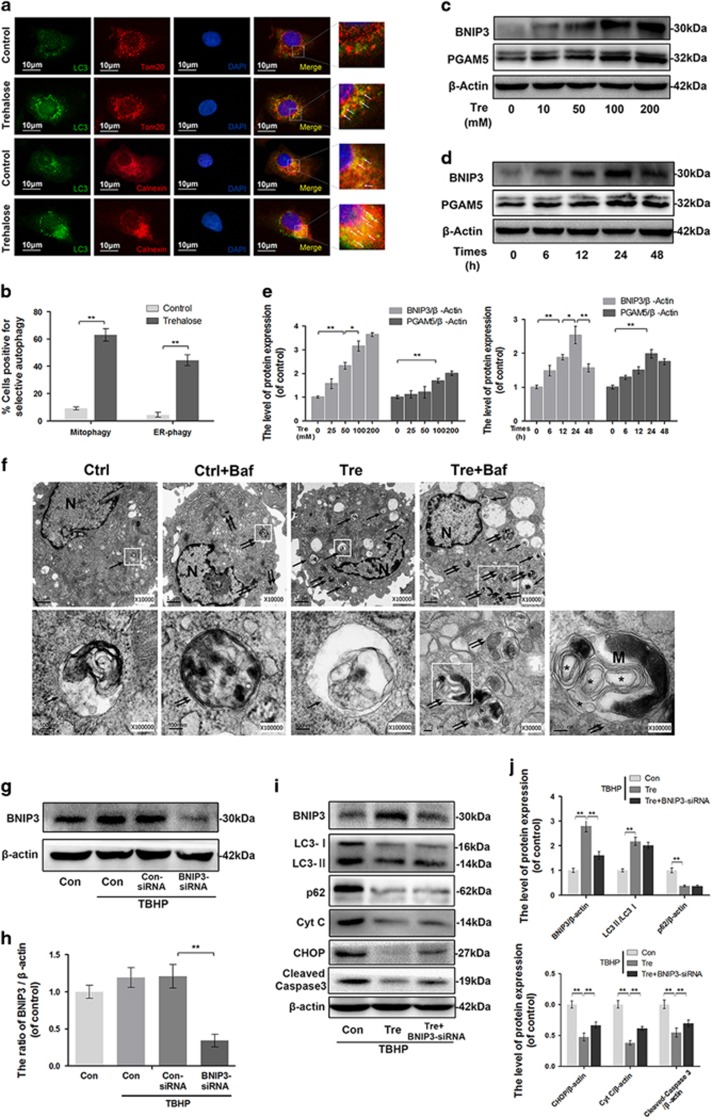
Trehalose induces mitophagy and reticulophagy in chondrocytes. (**a**) Mitophagy and reticulophagy were determined by assessing the co-localization (white arrow) between LC3-positive autophagosomes (green) and Tom20-labeled mitochondria (red) or calnexin-labeled endoplasmic reticulum (red; bar: 10 *μ*m). (**b**) Quantitation of the number of cells positive for mitophagy and reticulophagy per field according to immunofluorescence staining. (**c**–**e**) The protein levels of BNIP3 and PGAM5 in chondrocytes treated with trehalose as indicated. (**f**) TEM images of autophagic vesicles and selective autophagy in chondrocytes treated as indicated (× 10 000/ × 100 000). (Double arrow: autophagosome with double membrane structure; single arrow: autophagolysosome with single membrane structure; ‘M’: mitochondria; Asterisk: parts of ER whorls). (**g** and **h**) The protein level of BNIP3 in chondrocytes treated as indicated. (**i** and **j**) The protein levels of BNIP3, LC3, p62, Cyt C, CHOP and cleaved caspase 3 in chondrocytes treated as indicated. All data represent mean±S.D. (*n*=5). **P<0.01, *P<0.05

To determine whether trehalose could activate autophagy targeted in the mitochondria and ER, double-labeled immunofluorescence staining was performed. The results showed that LC3 co-localized with Tom20 (a mitochondria marker) and calnexin (an ER marker) after trehalose treatment, compared to control group (Figures 3a and b). PGAM5 and BNIP3 levels increased after trehalose treatment in a concentration- and time-dependent manner (Figures 3c–e). Furthermore, we observed autophagosomes, autophagolysosomes and selective autophagy by transmission electron microscopy (TEM). Bafilomycin A1 (Baf) was used to inhibit the fusion of autophagosome and lysosome and provide better view of the autophagosomes and contents. Compared to control group, trehalose-treated group showed more autophagosomes and autophagolysosomes formation in the cytoplasm (Figure 3f). Meanwhile, zoom up views revealed that the autophagosomes contained parts of ER whorls (some rough ER parts with ribosomes adhesion) and compact mitochondria in cells treated with trehalose plus bafilomycin A1 (Figure 3f). Furthermore, we explored the role of BNIP3 in trehalose-induced selective autophagy. Double-labeled immunofluorescence analysis showed the occurrence of BNIP3 positive points and the co-localization of BNIP3 with TOM20 and Calnexin in trehalose-treated group (Supplementary Figure S3). In addition, BNIP3 knockdown did not significantly change the expression levels of autophagy proteins but partially inhibited trehalose induced anti-apoptotic effect (Figures 3g–j). These data indicate that BNIP3-induced selective autophagy is responsible for anti-apoptotic effect of trehalose.

### Trehalose ameliorates TBHP-induced mitochondria dysfunction by enhancing autophagic flux

**Figure 4:**
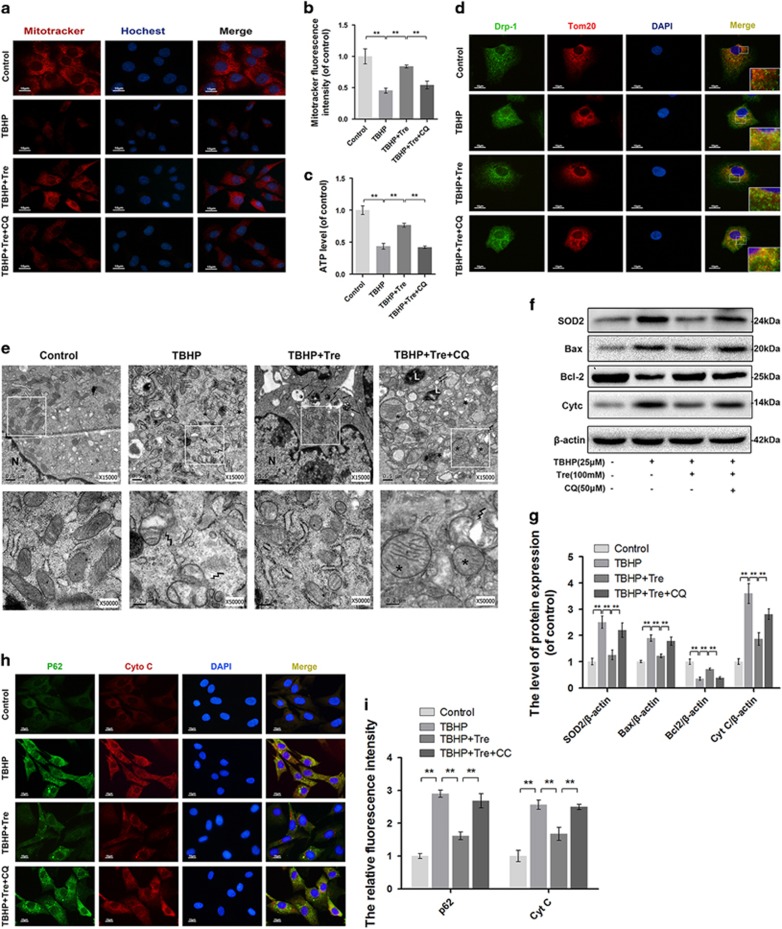
Trehalose promotes autophagic flux and attenuates mitochondrial dysfunction in chondrocytes. (**a** and **b**) Mitochondrial membrane potential was detected using Mitotracker and the nuclei were stained with Hoechst. (bar: 10 *μ*m). (**c**) ATP content was assessed by ATP-Glo Bioluminometric Cell Viability Assay. (**d**) Immunofluorescence double-labeled staining for co-localization of Drp-1 with Tom20 (Green: drp-1, red: Tom 20, bar: 10 *μ*m). (**e**) TEM images of the mitochondria and autophaghic change in chondrocytes (× 15 000 or 50 000). Lightning mark: mitochondrial fission; Asterisk: swollen mitochondira with fractured cristae; Double arrow: autophagosome with double membrane structure; single arrow: autophagolysosome). (**f** and **g**) The protein levels of SOD2, Bax, Bcl-2 and Cyt C in chondrocytes treated as indicated. (**h** and **i**) Double-labeled immunofluorescence staining of p62 and Cyt C in chondrocytes. (Green: p62, red: Cyt C, bar: 10 *μ*m). All data represent mean±S.D (*n*=5). **P<0.01

To determine whether the effects of trehalose on mitochondria dysfunction were related to autophagy-lysosome pathway, we examined the mitochondrial transmembrane potential in chondrocytes using the membrane potential-dependent mitochondria staining assay, MitoTracker. Treatment with trehalose markedly increased the fluorescence intensity compared to TBHP alone treated cells, but this was abrogated when autophagic flux was blocked by CQ (Figures 4a and b). ATP assay showed that TBHP treatment increased cellular ATP depletion, while trehalose pretreatment partially restored cellular ATP level in TBHP treated cells. The effect of trehalose on cellular ATP level was abolished by CQ (Figure 4c). Next, we performed double-labeled staining of the mitochondria fission proteins Drp-1 and Tom20. Drp-1 was distributed in the cytoplasm without localization in the mitochondria under normal condition, but co-localized with Tom20 after TBHP stimulation. Thus TBHP caused the translocation of Drp-1 into the mitochondria. Trehalose pretreatment inhibited mitochondria fission induced by TBHP, but this effect was blocked by CQ (Figure 4d). Electron microscopy revealed that the mitochondria appeared swollen, dissolved and fission in TBHP-treated chondrocytes. Pretreatment with trehalose recovered the morphology of the mitochondria with amounts of autophagolysosomes formation, but these effects were blocked by CQ (Figure 4e). Western blot analysis showed that TBHP significantly decreased B-cell lymphoma-2 (Bcl-2) level while increased superoxide dismutases 2 (SOD2), Bcl-2 Associated X Protein (Bax) and Cyt C levels. Treatment with trehalose attenuated the changes in SOD2, Bax, Cyt C and Bcl-2 levels induced by TBHP, while CQ reversed the effects of trehalose on the levels of SOD2, Bax, Cyt C and Bcl-2 (Figures 4f and g). Double-labeled immunocytochemical analysis showed similar changes in Cyt C and p62 staining in the chondrocytes subjected to different treatments (Figure 4h and i).

### Trehalose ameliorates TBHP-induced ER stress by enhancing autophagic flux

**Figure 5:**
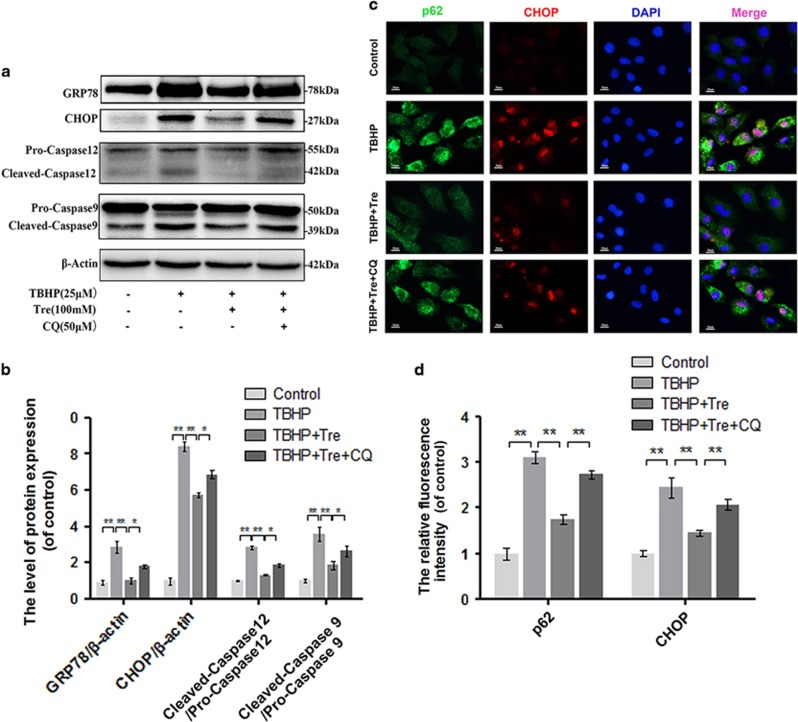
Trehalose relieves oxidative stress induced ER stress in chondrocytes. (**a** and **b**) The protein levels of GRP78, CHOP, Caspase 12 and Caspase 9 in chondrocytes were detected. (**c** and **d**) Double immunofluorescence staining of p62 and CHOP in chondrocytes. (Green: p62, red: CHOP, bar: 10 *μ*m). All data represent mean±S.D. (*n*=5). **P<0.01, *P<0.05

To determine whether autophagic flux was involved in the anti-ER stress effects of trehalose, ER stress markers were assessed by western blot analysis. We found significant increases in the levels of GRP-78, CHOP, cleaved caspase 12, and cleaved caspase 9 in TBHP-treated group compared to control group. Pretreatment with trehalose decreased the levels of these ER stress markers, while CQ reversed the effects of trehalose on these ER stress markers (Figures 5a and b). Double-labeled immunocytochemical analysis showed similar changes in ER stress marker CHOP and p62 staining in the chondrocytes subjected to different treatments (Figures 5c and d).

### Trehalose inhibits TBHP-induced apoptosis by enhancing autophagic flux

**Figure 6:**
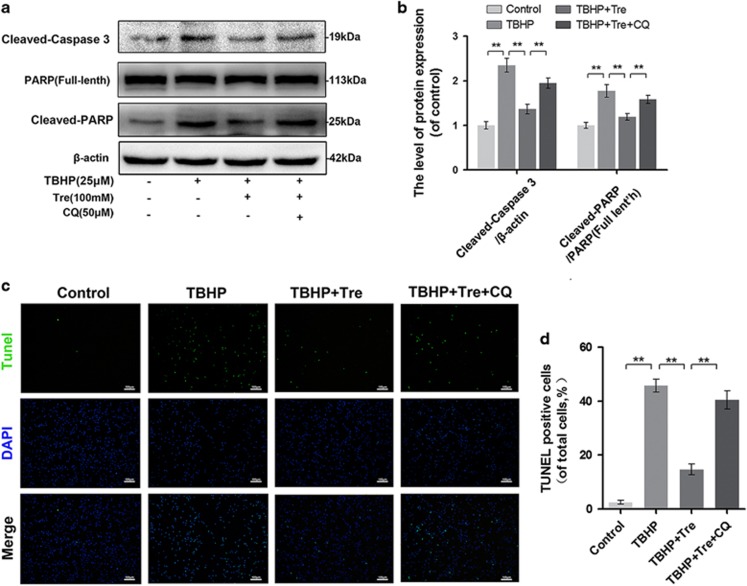
Trehalose inhibits TBHP induced apoptosis in chondrocytes. (**a** and **b**) The protein levels of cleaved caspase 3, PARP and cleaved PARP in chondrocytes treated as indicated were detected. (**c** and **d**) TUNEL assay of apoptotic chondrocytes (bar: 100 *μ*m). All data represent mean±S.D (*n*=5). **P<0.01

To determine whether apoptosis is attenuated by trehalose-induced autophagic flux activation, we examined the levels of cleaved caspase-3 and PARP by western blot analysis (Figures 6a and b) and DNA damage using the tunel method (Figure 6c and d). The results showed that trehalose inhibited TBHP-induced cleavage of caspase-3 and PARP, and rescued DNA damage in the chondrocytes. The anti-apoptotic effects of trehalose were blocked by CQ.

### Trehalose activates AMPK-ULK1 pathway but not mTOR-dependent pathway in chondrocytes

**Figure 7:**
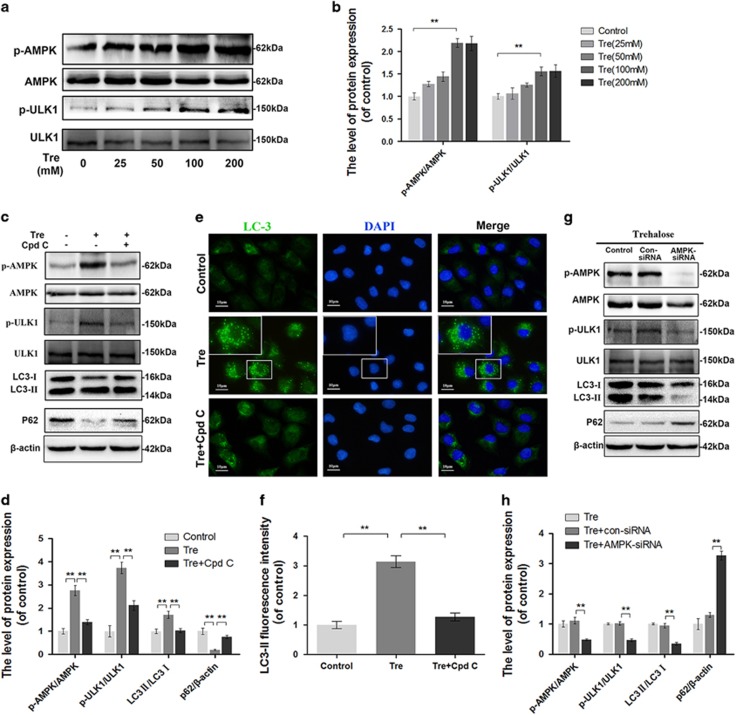
Trehalose promotes autophagic flux via activating AMPK-ULK1 pathway (**a** and **b**) The protein levels of p-AMPK, AMPK, p-ULK1 and ULK1 in chondrocytes treated with 0, 25, 50, 100 or 200 *μ*M Trehalose for 24 h. (**c** and **d**) The protein levels of p-AMPK, AMPK, p-ULK1, ULK1, LC3 and p62 in chondrocytes treated as indicated. (**e** and **f**) Immunofluorescence staining of LC3 (green) and the nucleus (blue). (bar: 10 *μ*m). (**g** and **h**) The protein levels of p-AMPK, AMPK, p-ULK1, ULK1, LC3 and p62 in chondrocytes treated with trehalose alone, trehalose combined with con-siRNA, or trehalose combined with AMPK-siRNA. All data represent mean±S.D. (*n*=5). **P<0.01

Western blot analysis showed no change in the expression levels of several proteins involved in mTOR-dependent pathway such as AKT, mTOR, and p70S6K after trehalose treatment (Supplementary Figure S4). However, trehalose significantly increased the phosphorylation of AMPK and ULK1 (Figures 7a and b). To further examine the role of AMPK in trehalose-induced autophagy, the chondrocytes were treated with AMPK inhibitor compound C (Cpd C) or transfected with AMPK-siRNA prior to trehalose treatment. Both Cpd C and AMPK-siRNA inhibited trehalose-induced AMPK-ULK1 pathway activation, LC3-II expression and p62 degradation (Figures 7c, d, g and h). Immunofluorescence analysis confirmed that trehalose upregulated LC3-II and this could be antagonized by Cpd C (Figures 7e and f). Meanwhile, we compared the activation of AMPK, mTOR and ULK1 in cells treated with trehalose and rapamycin (Supplementary Figure S5).

### Trehalose ameliorates OA development in mouse model

**Figure 8:**
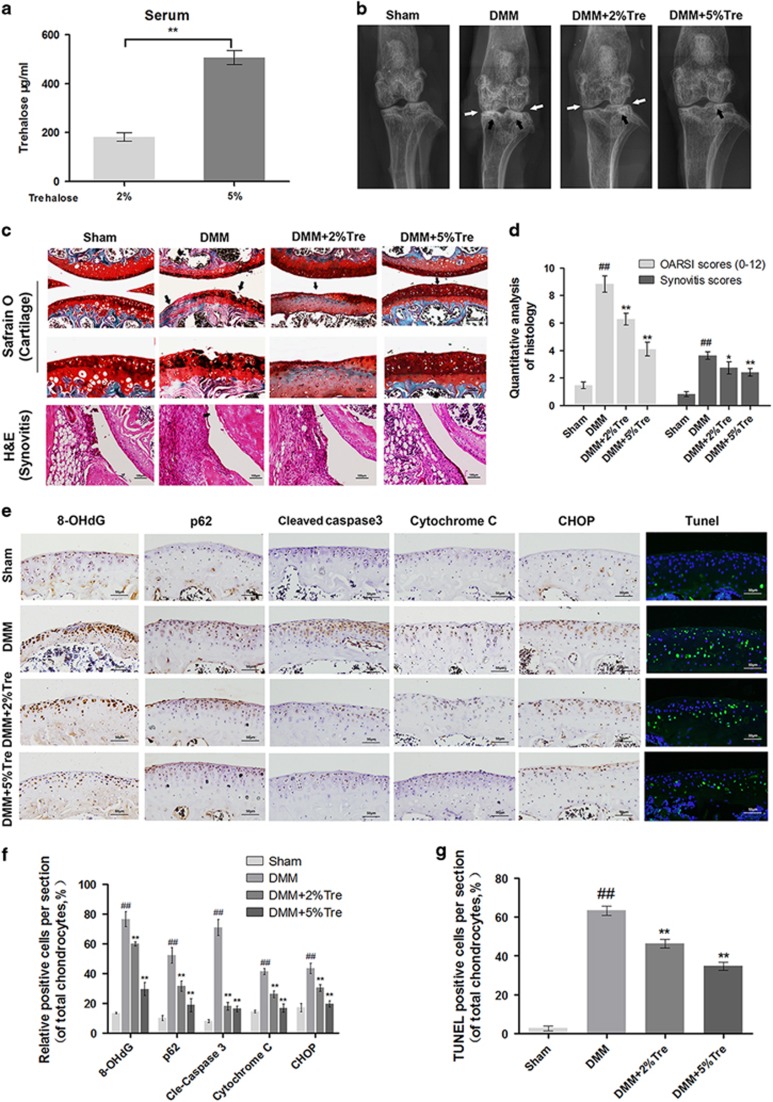
Trehalose ameliorates OA in mouse DMM model. (**a**) Serum levels of trehalose in DMM mice treated with 2% or 5% trehalose. (**b**) Digital X-ray images of knee joints in four groups. Narrowing of joint space was indicated by white arrows, and the calcification of cartilage surface was indicated by black arrows. (**c**) Representative S-O staining of cartilage and H&E staining of synovitis in four groups at 8 week post-surgery (bar: 100 or 50 *μ*m). (**d**) OARIS scores of cartilage and the scores of synovitis in four groups. (**e**) Immunohistochemical staining of 8-OHdG, p62, cleaved caspase 3, cytochrome *C* and CHOP and TUNEL staining in the mouse cartilage (bar: 50 *μ*m). (**f** and **g**) Quantitation of immunohistochemical and TUNEL staining. All data represent mean±S.D. (*n*=5). ^##^*P*<0.01, *versus* Sham, **P<0.01, *P<0.05, *versus* DMM

To investigate protective effects of trehalose on OA *in vivo*, we established a surgically induced DMM mouse model of OA. Oral administration of trehalose in the mice for 8 weeks led to significantly higher serum level of trehalose in 5% trehalose-treated mice compared with 2% trehalose-treated mice (Figure 8a). X-ray revealed that OA group presented aberrant narrowing of the joint space and increased cartilage surface density compared to sham group. Nevertheless, calcification of cartilage surface was milder in trehalose treated group in a dose-dependent manner (Figure 8b). Histological analysis by Safranin O and hematoxylin and eosin (H&E) staining showed that the cartilage surface was smooth and stained red in sham group, but we observed cartilage erosion, apparent hypocellularity, and massive proteoglycan loss in OA group. However, trehalose treated group exhibited less proteoglycan loss than OA group in a dose-dependent manner (Figure 8c). OARSI score of OA group was markedly higher (9.13±1.46) than that of sham group (1.25±0.20), while trehalose treated group had significantly lower OARSI scores (2% trehalose: 6.20±1.06; 5% trehalose: 4.11±1.36) than OA group. In addition, synovial thickening and hypercellularity were observed in OA group, while trehalose treatment alleviated synovitis compared to OA group (Figure 8d).

### Trehalose promotes autophagic flux and attenuates apoptosis of chondrocytes *in* v*ivo*

To demonstrate the mechanism of the chondroprotective effects of trehalose *in vivo*, we performed immunohistochemical staining of 8-hydroxy-2′-deoxyguanosine (8-OHdG, marker of oxidative stress), p62, Cyt C, CHOP and cleaved caspase 3. The cartilage of OA group showed elevated positive staining for 8-OHdG, p62, Cyt C, CHOP and cleaved caspase 3 compared to sham group while trehalose administration markedly decreased the staining of these proteins in a dose dependent manner (Figures 8e and f). In addition, TUNEL assay showed significantly higher number of apoptotic chondrocytes in OA group compared to sham group, while trehalose administration decreased the number of apoptotic chondrocytes in a dose-dependent manner (Figures 8e and g).

## Discussion

Growing evidence supports that autophagy and apoptosis are involved in the pathogenesis of OA.^18, 33, 34^ However, the role of autophagic flux in OA development has not been fully elucidated. SQSTM1/p62 protein has been used to monitor autophagic flux changes becuase it is a linkage molecule between LC3-decorated autophagosomes and ubiquitin-conjugated protein aggregates that are ultimately degraded in autolysosomes.^14^ Our study demonstrated the accumulation of p62 in the cartilage and chondrocytes derived from patients with severe OA compared to controls. The change of p62 expression was consistent with that of cleaved caspase 3, which indicated that disrupted autophagic flux might be associated with apoptosis in human OA chondrocytes. In addition, many *in vitro* studies have shown that chronic and sustained oxidative stress-induced apoptosis and disrupted autophagic flux.^35, 36, 37, 38, 39^ In this study, both p62 and cleaved caspase 3 levels increased under oxidative stress induced by TBHP in mouse chondrocytes. Interestingly, several previous studies suggested that ROS would initiate autophagy as a self-protective mechanism responding to transient oxidative stress.^34, 40^ However, oxidative stress-induced autophagic changes was dose- and time-dependent. OA as well as other degenerative diseases develop under chronic and sustained oxidative stress. Accordingly, we applied relative mild concentration of TBHP (25 *μ*M) to treat chondrocytes for total 24 h to model OA *in vitro*.

Cells exposed to oxidative stress show increased SOD level and the translocation of mitochondrial fission protein drp-1 from the cytoplasm to the mitochondria, resulting in fission and broken mitochondria.^41^ Oxidative stress allows the oligomerization of Bax, which disrupt the mitochondrial outer membrane.^8^ Consequently, apoptotic proteins such as Cyt C are released into the cytoplasm, triggering caspases activation.^3^ In present study, we found that pretreatment with trehalose significantly decreased SOD2, Bax and Cyt C while increased Bcl-2 in chondrocytes under oxidative stress. Moreover, trehalose reversed oxidative stress-mediated mitochondrial membrane potential collapse, ATP level decrease and drp-1 translocation. These findings indicate that the protective role of trehalose against chondrocytes apoptosis is related to the attenuation of mitochondrial dysfunction.

ER stress is a key regulator in the apoptotic process of OA pathogenesis.^8^ Moderate ER stress is an adaptive protective process in chondrocytes, but excessive ER stress triggers the cleavage of caspase 12 and induces apoptosis.^42^ GRP78 and CHOP are canonical markers for ER stress.^43, 44^ Our data showed that trehalose markedly reduced the levels of GRP78 and CHOP, indicating that trehalose inhibits oxidative stress-induced ER stress. In addition, both mitochondrial injury and ER stress converge towards activating a series of caspases and PARP followed by DNA damage.^3^ Our results showed that trehalose significantly decreased apoptotic chondrocytes and the levels of cleaved caspase 9, cleaved caspase 3 and cleaved PARP, which were upregulated by TBHP treatment. These results indicate that trehalose attenuates apoptosis of chondrocytes exposed to sustained oxidative stress.

To reveal the relationship between autophagy and apoptosis associated with mitochondria injury and ER stress, we applied CQ, a classic inhibitor that functions downstream of autophagy. The protective effects of trehalose were abrogated when autophagic flux was inhibited, indicating that trehalose plays protective role by enhancing autophagic flux. Selective autophagy has been recognized as a more effective and targeted way to remove damaged or otherwise unwanted organelles and ensure cell survival under stress.^45^ Several proteins have been reported as signaling molecules, including Pink1/Parkin, BNIP3/Nix, and PGAM5, for anchoring autophagic cargo in damaged organelles.^45, 46^ BNIP3 is localized in both the mitochondria and ER and interacts with LC3.^25^ PGAM5 is a mitochondria-localized phosphatase responsible for the dephosphorylation of FUN14 domain containing 1 (FUNDC1) at Ser13, which has high affinity to LC3. Dephosphorylated FUNDC1 increases the interaction between the mitochondria and LC3, leading to selective autophagic removal of the affected mitochondria.^46^ In our study, double-labeled immunofluorescence analysis suggested that trehalose promoted LC3 localization in the mitochondria and ER. Moreover, both BNIP3 and PGAM5 were upregulated by trehalose, while BNIP3 knockdown resulted in partial inhibition of trehalose induced anti-apoptotic effect but did not alter autophagy. These results suggest that the protective effects of trehalose for mitochondrial injury and ER stress associated apoptosis might be relation to the induction of BNIP3-mediated selective autophagy.

Trehalose is a novel autophagic inducer that functions via mTOR-independent pathway.^26, 27, 28, 30^ However, recent studies have revealed that trehalose enhances autophagic flux in hepatocytes via Glucose transporter (GLUT) inhibition, followed by the activation of AMPK-ULK1 pathway.^47, 48^ Our results are consistent with this idea because we found that trehalose-induced autophagy in chondrocytes was associated with the phosphorylation of AMPK, which directly activated ULK1, but not with mTOR-related signaling molecules such as AKT, mTOR and p70S6K. Due to the side effects of mTOR inhibition, AMPK-dependent pathway seems to be a safer choice for autophagy-related clinical applications. However, it remains unclear how trehalose promotes the phosphorylation of AMPK in chondrocytes and whether it is correlated with GLUT inhibition, and need further investigations.

OA progression involves the loss of proteoglycans on the cartilage surface, calcification changes, chondrocyte apoptosis and synovitis. Among these changes, decreased proteoglycans is the main characteristic of cartilage degeneration.^49^ DMM mouse model is a reliable OA model for *in vivo* studies.^50^ We found that trehalose significantly reduced OARSI score and the severity score of synovitis in DMM mice. In addition, trehalose-reduced apoptosis and decreased the levels of 8-OHdG, cleaved caspase 3, Cyt C, CHOP and p62, which indicated that promoting autophagic flux is a potential mechanism underlying the anti-apoptotic effect of trehalose *in vivo*.

In summary, we demonstrated that autophagic flux marker p62 and apoptosis marker cleaved caspase 3 were upregulated in human OA and TBHP-treated mouse chondrocytes. Trehalose administration targeted activated mitophagy and reticulophagy and restored oxidative stress-induced autophagic flux disruption in mouse chondrocytes, which was associated with the activation of AMPK-ULK1 but not mTOR-dependent pathway. Autophagic changes stimulated by trehalose protected chondrocytes against apoptosis by attenuating mitochondrial dysfunction and ER stress induced by TBHP. Further *in vivo* experiments in OA mice model confirmed the therapeutic efficacy of trehalose on OA. These findings suggest the potential of trehalose in the prevention and treatment of osteoarthritis.

## Materials and methods

### Ethics statement

Human articular cartilage tissue collection and the experiments involved in human cartilage tissue was approved by Ethical Committee of the Second Affiliated Hospital, Wenzhou Medical University and following the guidelines of the Declaration of Helsinki.^51^ The letter of ethics approval was provided in Supplementary File 1. All animal surgical interventions, treatments and postoperative care procedures were performed in strict accordance with the Animal Care and Use Committee of Wenzhou Medical University (wydw2014-0129).

### Reagents

D-(+)-Trehalose dehydrate (purity >99 %), toluidine blue, tert-Butyl hydroperoxide solution (TBHP), bafilomycin A1 (Baf), chloroquine (CQ) and type II collagenases were purchased from Sigma-Aldrich (St Louis, MO, USA). The primary antibody against p62, Tom20, BNIP3, PGAM5, Drp1, SOD2, PARP, cytochrome C, caspase 9, 8-0HdG and *β*-actin were from Abcam (Cambridge, UK), LC-3 antibody was from Sigma-Aldrich (St Louis, MO, USA). GRP78 and Calnexin antibodies were from Santa Cruz Biotechnology (Santa Cruz, CA, USA); Anti-CHOP, goat anti-rabbit, and anti-mouse IgG-HRP were from Bioworld (OH, USA) and antibodies against Atg3, Atg7, Atg12, Beclin1, p-AKT, AKT, p-mTOR, mTOR, p-p70S6K, p70S6K, p-AMPK, AMPK, p-ULK1, ULK1, ubquintin, caspase 12, Bcl-2, Bax and cleaved-caspase3 were from Cell Signaling (Danvers, MA, USA); Alexa Fluor488 labeled and Alexa Fluor594 labeled Goat Anti-Rabbit IgG (H+L) second antibody were from Jackson ImmunoResearch (West Grove, PA, USA). 4′,6-diamidino-2-phenylindole (DAPI) was from Beyotime (Shanghai, China).

### Human cartilage and chondrocytes culture

Control human articular cartilages from 5 donors with no significant clinical and imaging features of OA were obtained from femoral condyles and tibial plateaus at autopsy (43–75 years old; mean, 57.3 years; Kellgren-Lawrence grade,^52^ 0 or I; *n*=5), which were defined as relative normal. OA human articular cartilages were obtained form 9 patients (53–76 years old; mean, 60.1 years; Kellgren–Lawrence grade, III or IV; *n*=9) undergoing total knee arthroplasty (representative X-ray image was presented in Supplementary Figure S6). Cartilage tissues were cut into 5-*μ*m sagittal sections and embedded in paraffin for histological analysis. To obtain primary human chondrocytes, hyaline cartilage was cut into pieces and incubated with 2 mg/ml of collagenase II in DMEM/F12 at 37 °C for 4 h. After washing by PBS and resuspension, chondrocytes were cultured in a six-well plate at a seeding density of 2 × 10^5^ cells per ml in DMEM/F12 supplemented with 10% FBS and 1% antibiotic in 5% CO2 at 37 °C. Chondrocytes no later than first passage were used for the experiments.

### Primary mice chondrocytes culture

C57BL/6 mice (5 males and 5 females, 10 days old) were euthanized with sodium pentobarbital. The knee cartilages of mice were collected carefully under aseptic conditions and treated with 2 mg/ml (0.1%) collagenase II for 4 h at 37 °C. After washing by PBS and resuspension, mouse chondrocytes were cultured in DMEM/F12 supplemented with 10% FBS and 1% antibiotic in 5% CO_2_ at 37 °C. The second passage chondrocytes were identified by toluidine blue staining and immunostaining with collagen II. The second-passage chondrocytes were used for the experiments.

### Cell viability assay

The cytotoxicity of TBHP and trehalose on chondrocytes were detected with cell counting kit-8 (CCK-8; Dojindo Co, Kumamoto, Japan) according to the manufacturer’s protocol. Chondrocytes were seeded in 96-well plates (50 000 cell per cm^2^) and then treated with TBHP and/or trehalose. The cells were washed with phosphate-buffered saline (PBS), and then 10 *μ*l of CCK-8 solution was added to each well of the plate and incubated for 2 h at 37 °C. The absorbance of the wells was measured at 450 nm using a micro-plate reader. All experiments were repeated five times.

### Western blot analysis

The total protein was isolated from chondrocytes using RIPA lysis buffer with 1 mM phenylmethanesulfonyl fluoride followed by 15 min centrifugation at 12 000 r.p.m. at 4 °C. Protein concentration was measured using BCA protein assay kit (Beyotime). Forty nanogram of protein was separated by SDS-polyacrylamide gel electrophoresis and transferred to polyvinylidene difluoride membrane (Bio-Rad, Hercules, CA, USA). After blocking with 5% nonfat milk for 2 h, the membranes were incubated with primary antibody overnight at 4 °C, followed by incubation with secondary antibodies for 2 h at room temperature. After 3 times washing with TBST, the blots were visualized by electrochemiluminescence plus reagent (Invitrogen, Carlsbad, CA, USA), and analyzed Image Lab 3.0 software (Bio-Rad).

### Transmission electron microscopy

Mouse chondrocytes were fixed in 2.5% glutaraldehyde overnight, post fixed in 2% osmium tetroxide for 1 h and stained with 2% uranyl acetate for 1 h. After dehydration in a series of acetone, samples were embedded into araldite and cut into semi-thin sections, which were stained with toluidine blue to locate cell position and observed under a transmission electron microscope (Hitachi, Tokyo, Japan). For quantitative analysis of autophagosomes and autolysosomes, 25 fields of each section were randomly selected and captured at a final magnification of 25 000, and the number of autophagosomes and autolysosomes in each captured field was counted as describe dpreviosuly.^53^ Autophagosomes had double-membrane structures which contained fractured organelles or non-functional macromolecules. Autolysosomes had only one limiting membrane and contained cytoplasmic material and /or organelles at various stages of degradation.

### Immunofluorescence

Chondrocytes were rinsed in PBS, fixed in 4% paraformaldehyde and permeated in 0.1% Triton X-100 for 15 min. Then the cells were blocked with 5% bovine serum albumin for 1 h at 37 °C, rinsed with PBS and incubated with primary antibodies in a humid chamber overnight at 4 °C. The cells were washed and incubated with Alexa Fluor488 or Alexa Fluor594 conjugated second antibodies for 1 h at room temperature and labeled with DAPI for 5 min. Twenty-five fields of each slides were chosen randomly for observation with a fluorescence microscope (Olympus Inc., Tokyo, Japan), and fluorescence intensity was measured using Image J software 2.1 (Bethesda, MD, USA). Cells positive for mitophagy and ER-phagy were defined as the cells with co-localization of LC3 and Tom20 as well as Calnexin.

### MMP Assay

The mitochondrial transmembrane potential (MMP) was assessed by using MitoTrackerRed CMXRos (Molecular Probes, Thermo). Chondrocytes were stained with 50 nM MitoTrackerRed for 30 min at 37 °C, and nuclei were stained with Hoechst 33258 for 10 min. Red fluorescence images of at least 25 random microscopic fields were acquired per slide for microscopic observation with a fluorescence microscope (Olympus Inc., Tokyo, Japan), and fluorescence intensity was measured using Image J software 2.1 (Bethesda, MD, USA).

### ATP Assay

The ATP-Glo Bioluminometric Cell Viability Assay (Biotium, Hayward, CA, USA) was used to assess cellular ATP levels according to the manufacturer’s protocol. Data were collected from five replicate wells for each experiment.

### TUNEL

Chondrocytes or cartilage sections were fixed and then stained with *in situ* cell death detection kit (Roche, Basel, Switzerland) according to the manufacturer’s instructions for 30 min at 37 °C and the nuclei was stained with DAPI. Twenty-five fields of each slide were randomly selected and captured under a fluorescence microscope (Olympus) to count TUNEL positive cells.

### siRNA transfection

siRNA for mice BNIP3 and AMPK gene was designed and synthesized (RiboBio, Guangzhou, China) with the following sequence: BNIP3-siRNBA: 5′-CAGCCUCCGUCUCUAUUUATTT-3′ AMPK-siRNA: 5′-CGTCATTGATGATGAGGCT-3′. Chondrocytes were seeded in a six-well plate and cultured for 24 h to 60–70% confluency. The cells were transfected with 50 nM negative control or siRNA duplexes using Lipofectamine 2000 siRNA transfection reagent (Thermo Fisher, UT, USA).

### Animal model

Sixty 10-week-old C57BL/6 male mice were purchased from Animal Center of Chinese Academy of Sciences (Shanghai, China). Osteoarthritis was induced by surgical destabilization of the medial meniscus (DMM) as previously described.^50^ Arthrotomy without the transaction of medial meniscotibial ligament was performed in the left knee joint of mice and the joint was used as sham group. After surgery, the mice were randomly divided into four groups: sham group, DMM group, DMM plus 2% trehalose treatment group, DMM plus 5% trehalose treatment group. 2 or 5% (g/vol (ml)) trehalose was given to DMM mice in drinking water, starting at the day after surgery and continuing until the day the mice died. The concentration of trehalose in mice serum after 8 weeks of trehalose administration was determined using the Kit from Solarbio (Beijing, China). After 8 weeks of surgery, X-ray imaging was performed on all mice to evaluate the joint space and calcification changes of cartilage surface using a digital X-ray machine (Kubtec Model XPERT.8; KUB Technologies Inc.).

### Histopathologic analysis

The mice were killed by intraperitoneal injection of 10% chloral hydrate and the knee joint were collected 8 weeks after surgery. The specimens were fixed in 4% (v/v) paraformaldehyde for 24 h and decalcified in 10% (v/v) EDTA for 1 month. The tissues were dehydrated, embedded in paraffin and cut into 5-*μ*m sagittal sections. Slides of each joint were stained with safranin O-fast green (S-O) and hematoxylin and eosin (H&E). Fifteen mice in each group were evaluated by Osteoarthritis Research Society International (OARSI) scoring system for medial femoral condyle and medial tibial plateau as described previously.^54^ The severity of synovitis was graded using a scoring system as previously described:^49^ Enlargement of the synovial lining cell layer on a scale of 0–3 (0=1–2 cells,1=2–4 cells, 2=4–9 cells and 3=10 or more cells) and density of cells in the synovial stroma on a scale of 0–3 (0=normal cellularity, 1=slightly increased cellularity, 2=moderately increased cellularity and 3=greatly increased cellularity).

### Immunohistochemical analysis

The sections embedded in paraffin were deparaffinized and rehydrated and endogenous peroxidase was blocked by 3% hydrogen peroxide. The sections were incubated with 0.4% pepsin (Sangon Biotech, Shanghai, China) in 5 mM HCl at 37 °C for 20 min for antigen retrieval. The sections were incubated with 5% bovine serum albumin for 30 min at room temperature, then with primary antibody overnight at 4 °C, and finally with HRP-conjugated secondary antibody. Images were analyzed by Image-Pro Plus software, version 6.0 (Media Cybernetics, Rockville, MD, USA). Five mice of each group were used for quantitative analysis.

### Statistical analysis

The results were presented as mean±S.D. Statistical analyses were performed using SPSS statistical software program 20.0 (IBM, Armonk, NY, USA). Data were analyzed by one-way analysis of variance (ANOVA) followed by Tukey’s test for comparison between control and treatment groups. Nonparametric data (OARSI scores and synovitis scores) were analyzed by the Kruskal–Wallis H test. *P* <0.05 was considered significant.

## Publisher’s Note:

Springer Nature remains neutral with regard to jurisdictional claims in published maps and institutional affiliations.
