# Supplementary figures and images for: Trehalose ameliorates oxidative stress-mediated mitochondrial dysfunction and ER stress via selective autophagy stimulation and autophagic flux restoration in osteoarthritis development
Source: Cell Death Dis. 2017 Oct 5;8(10):e3081–. doi: 10.1038/cddis.2017.453 (PMC5680575; doi:10.1038/cddis.2017.453)

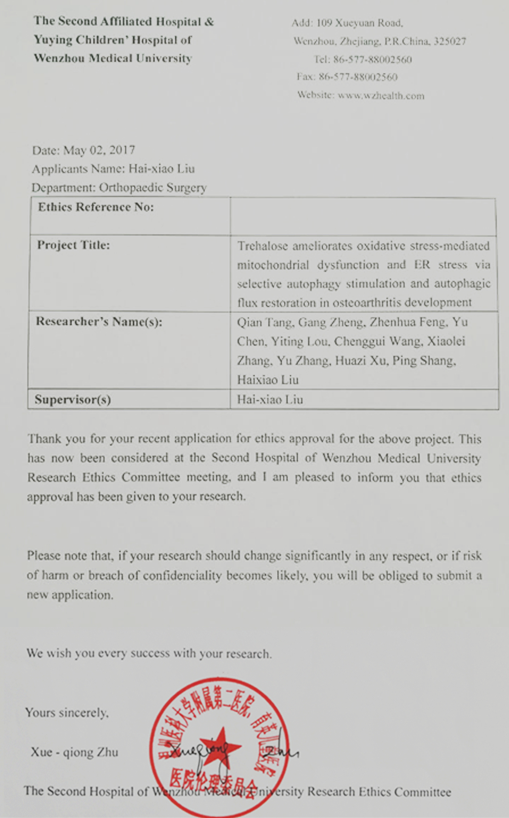

Supplement: Supplementary file 1 [file cddis2017453x1.png]

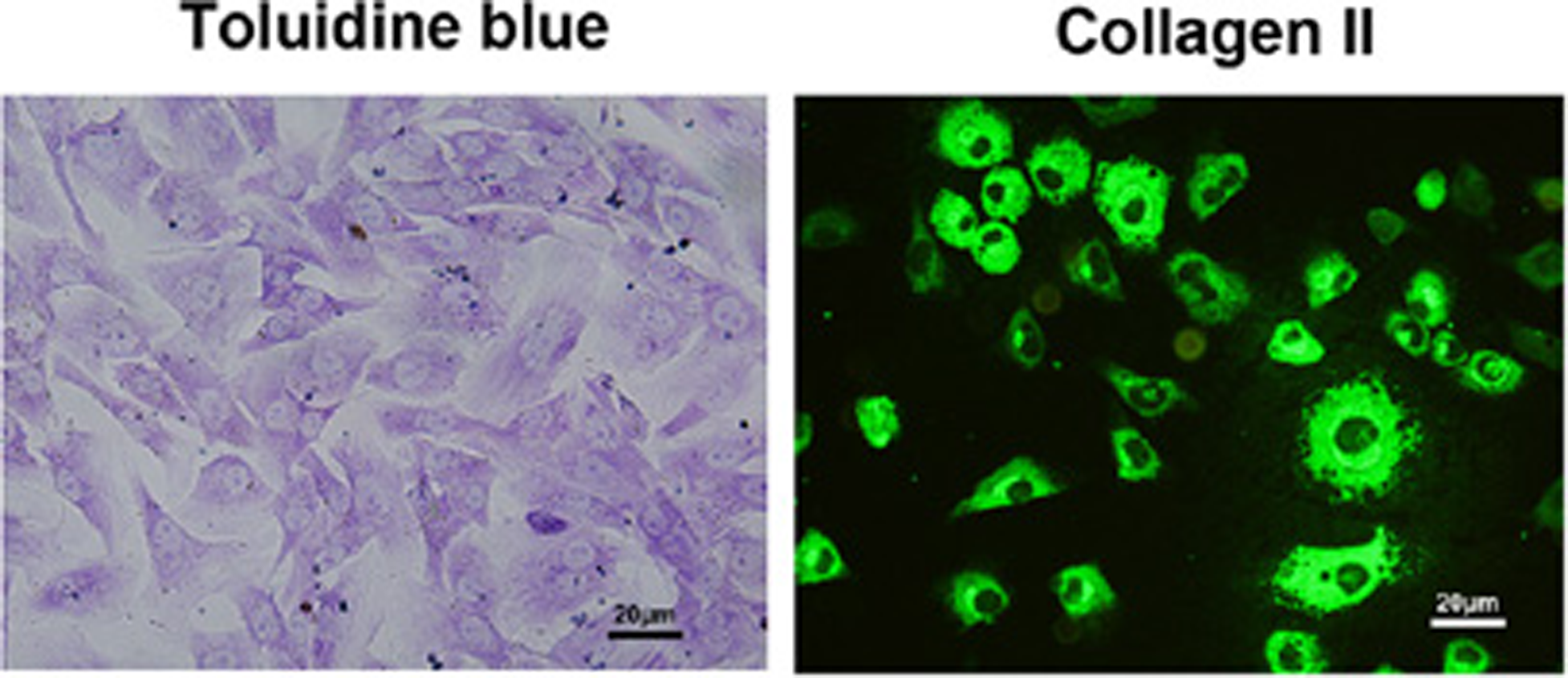

Supplement: Supplementary Figure [file cddis2017453x2.tif]

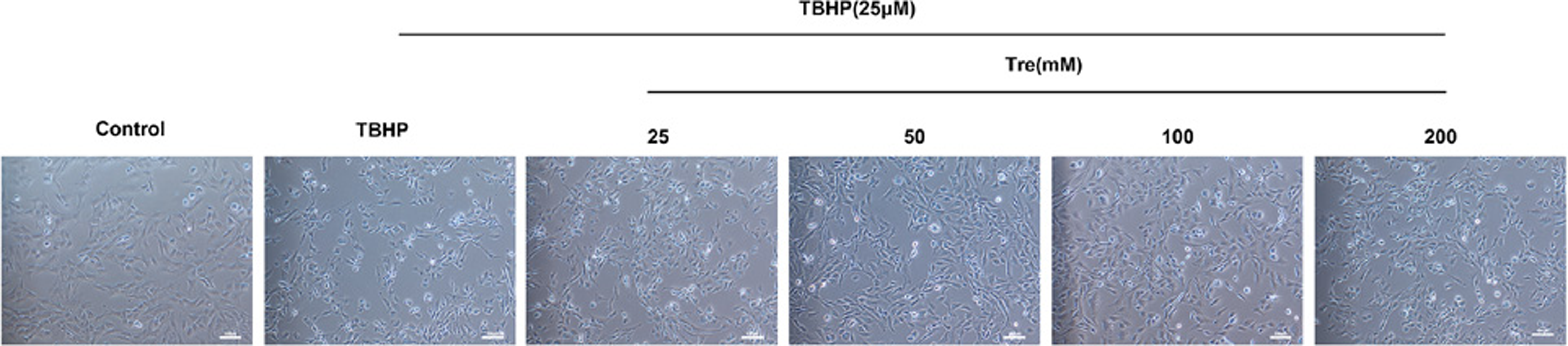

Supplement: Supplementary Figure [file cddis2017453x3.tif]

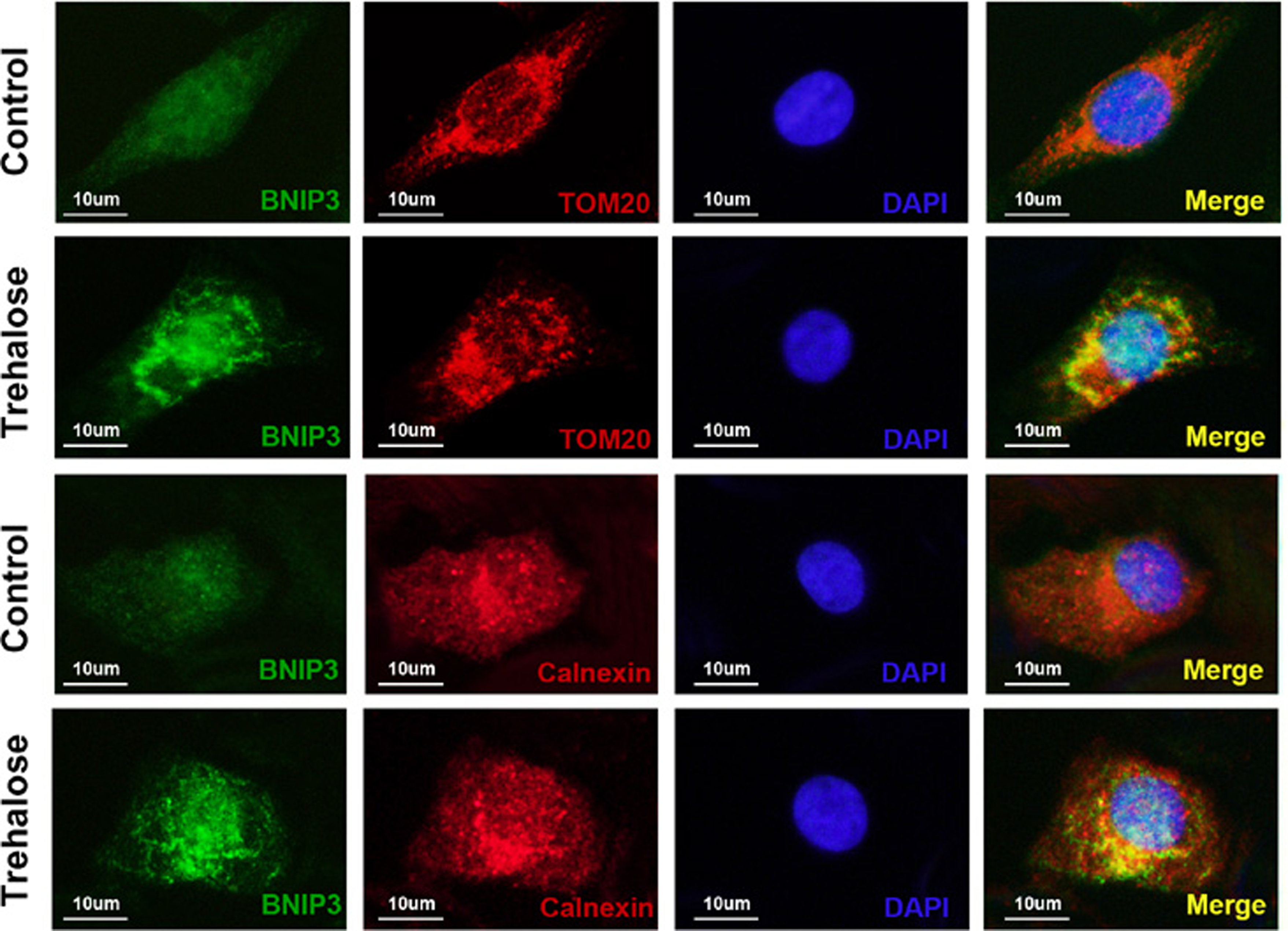

Supplement: Supplementary Figure [file cddis2017453x4.tif]

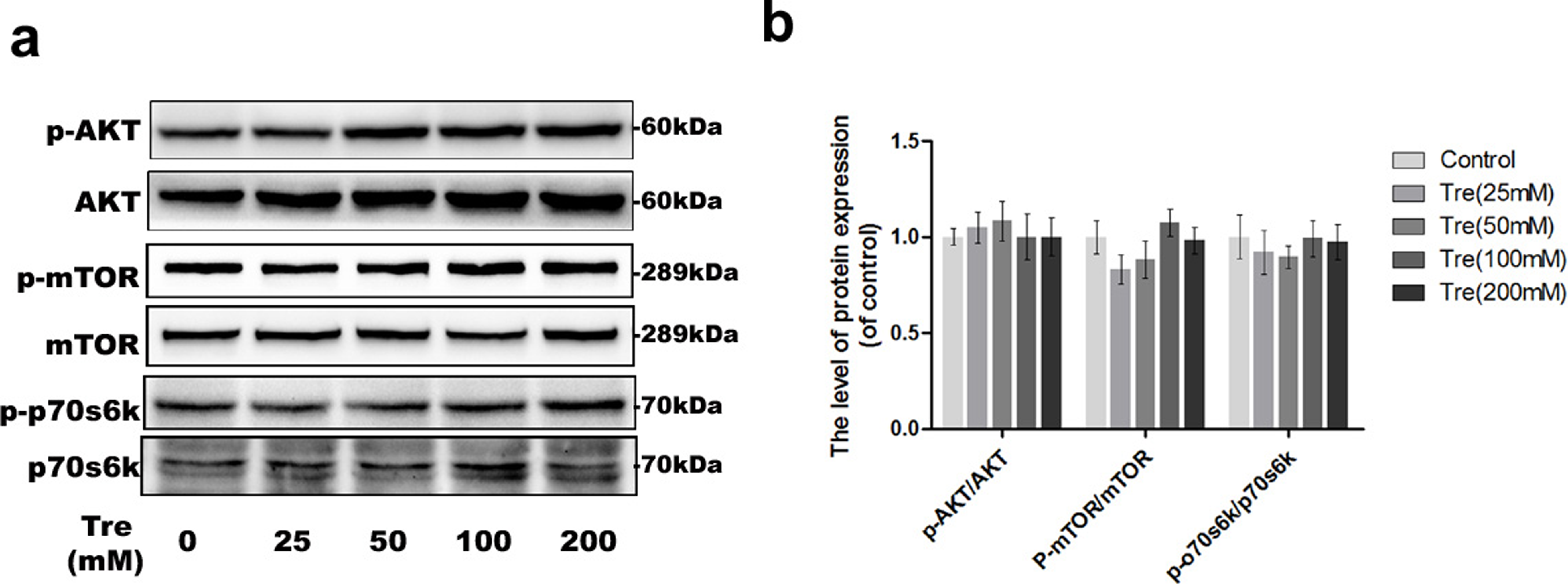

Supplement: Supplementary Figure [file cddis2017453x5.tif]

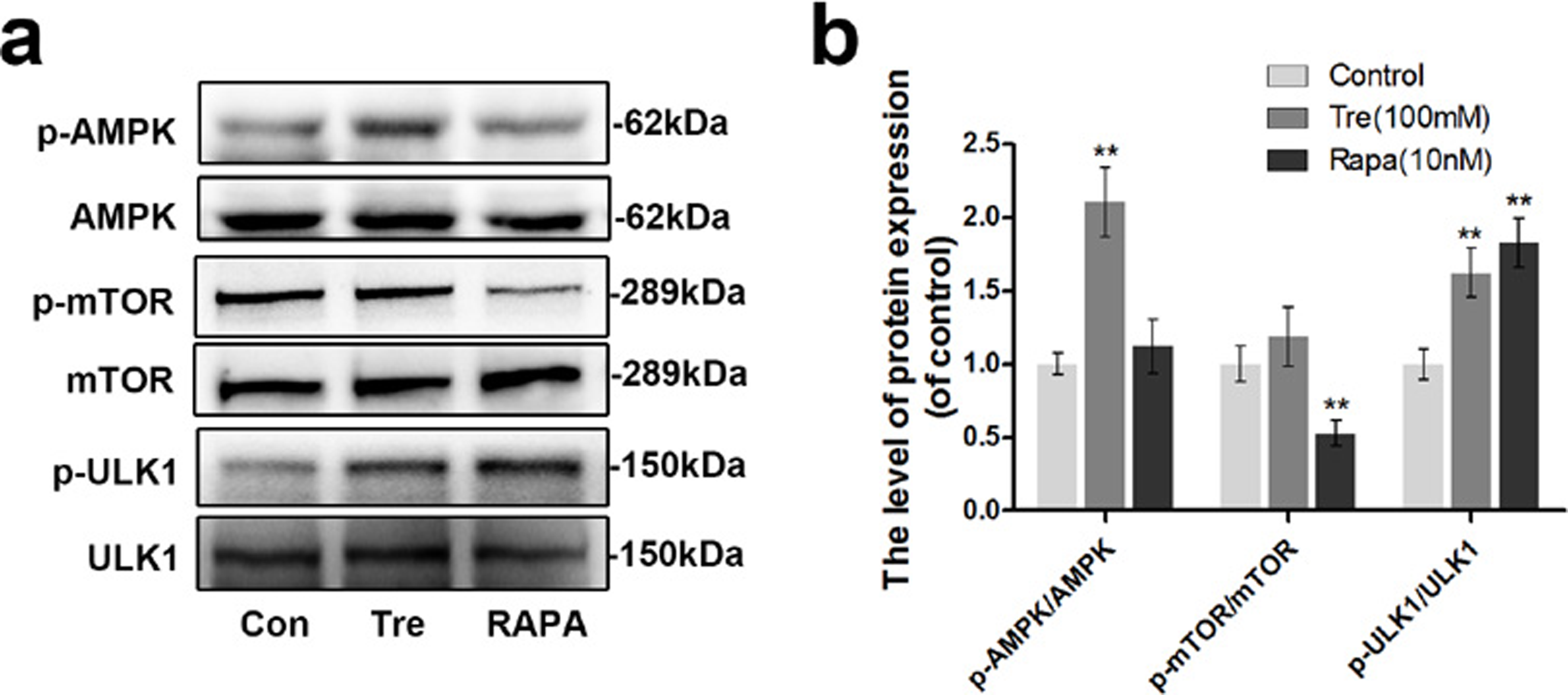

Supplement: Supplementary Figure [file cddis2017453x6.tif]

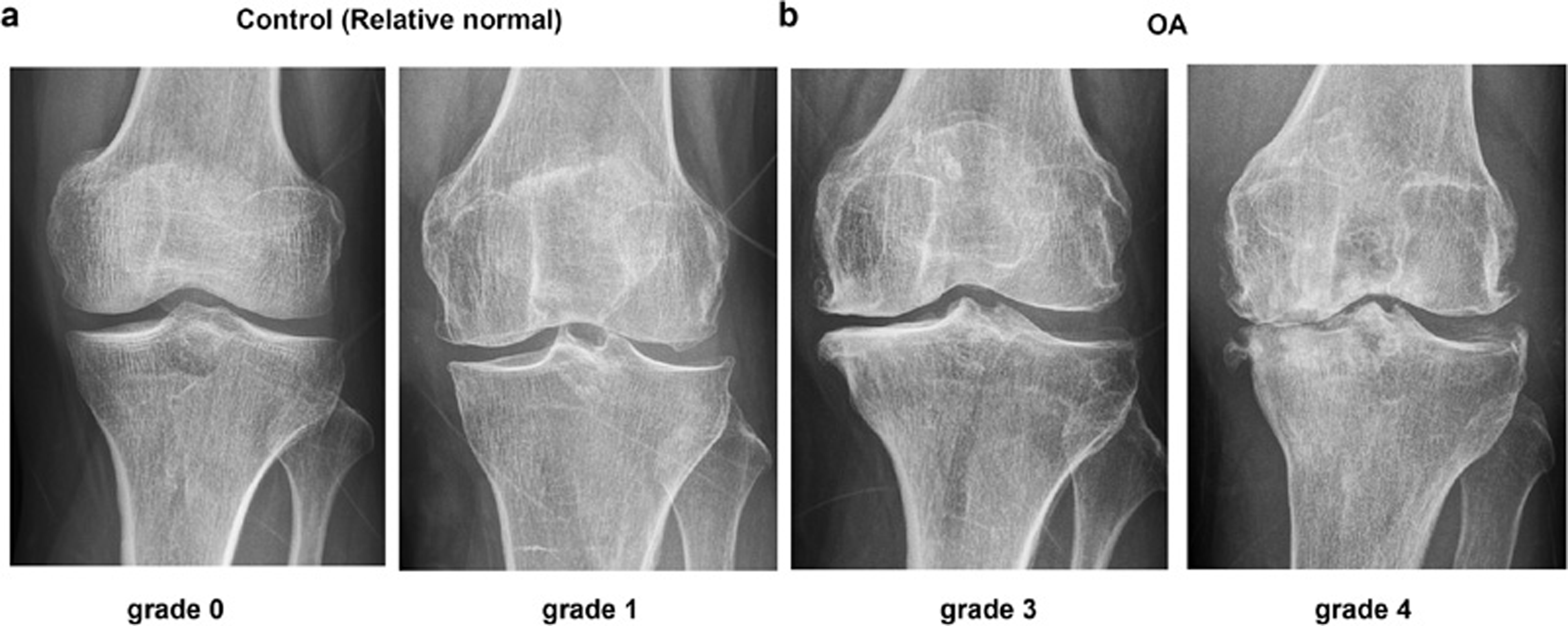

Supplement: Supplementary Figure [file cddis2017453x7.tif]

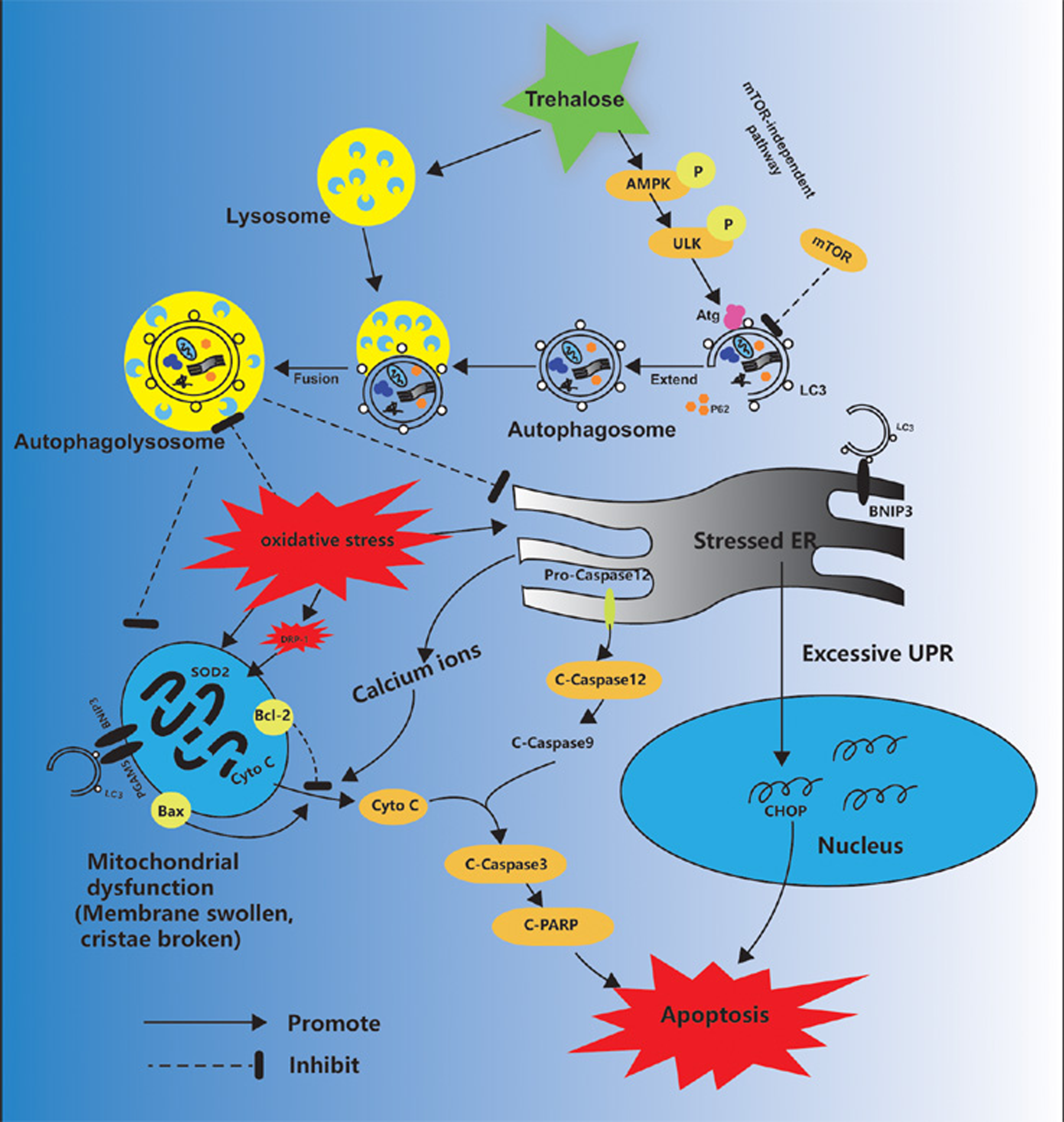

Supplement: Supplementary Figure [file cddis2017453x8.tif]
